# Perineal papilliferous syringocystadenoma: An unusual localization

**DOI:** 10.1016/j.ijscr.2023.109135

**Published:** 2023-12-10

**Authors:** Hamza Ouzzaouit, Boubker Idrissi Kaitouni, Sekkat Hamza, Fouad Zouaidia, Yassine Berrada, Farid Sabbah

**Affiliations:** a Digestive Surgical Department C, Ibn Sina University Hospital, Rabat, Morocco; b Faculty of Medicine and Pharmacy, Mohammed V University in Rabat, Morocco; c Anatomopathology Department, Ibn Sina University Hospital Rabat, Morocco; d Dermatology Department, Ibn Sina University Hospital Rabat, Morocco

**Keywords:** Cystic masse, Syringocystadenoma papiliferum, Perineal, Surgery

## Abstract

**Introduction and importance:**

Syringocystadenoma papiliferum (SCAP) is an infrequent, benign neoplasm originating from the apocrine or, less frequently, eccrine sweat glands. This condition predominantly manifests in regions such as the head, face, neck, and trunk. Notably, it is frequently associated with hamartomas of endocrine, sebaceous, or follicular origin, as well as with sebaceous nevi.

**Case presentation:**

In the context of this study, we present a clinical case involving a 65-year-old patient who exhibited an atypical anatomical presentation of syringocystadenoma papiliferum.

**Clinical discussion:**

This case highlights the importance of considering SCAP as a potential diagnosis of perineal cystic masses that need a surgical excision due to the risk of malignancy transformation.

**Conclusion:**

The uniqueness of the case under consideration lies in the rarity of syringocystadenoma papiliferum (SCAP), the atypical perineal localization, and its occurrence at an advanced age (65 years).

## Highlights

• Papilliferous syringocystadenoma (SCAP) is a very rare entity.
• Anoperineal localization of a SCAP is extremely rare.
• Surgical excision is the mainstay treatment in SCAP due to the risk of malignant degeneration.

## 1 Introduction

Syringocystadenoma papiliferum (SCAP) represents a rare benign adnexal neoplasm that can manifest either sporadically or as a secondary tumor within the context of Jadassohn's sebaceous nevus [1]. While the most commonly affected anatomical site is the head and neck region [1], occurrences on the trunk and extremities, though less frequent, have been documented. In rare instances, lesions have presented in areas such as the groin, buttocks, and the anogenital region [2,3].

SCAP typically exhibits an *exo*-endophytic configuration, often characterized by a crater-like morphology and a papillary architectural pattern. This papillary structure consists of double-layered tubular formations composed of luminal cells with a cuboidal to columellar appearance, frequently demonstrating apocrine secretion. These luminal cells are enveloped by a peripheral layer of basal/myoepithelial cells [3].

By using the 2020 Scare model [4], we report a case of a patient presenting a perineal syringocystadenoma papiliferum treated by a surgery excision. A literature review was carried on.

**Fig. 1:**
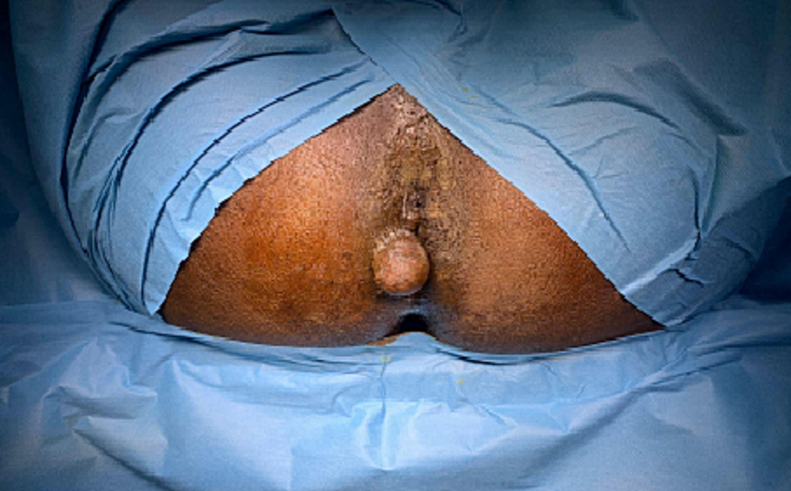
Proctological examination of the patient A: Sagital view of pelvis B: Coronal view of pelvis.

Presentation of case:

A 65-year-old male patient, devoid of prior medical history, presented with a right latero-anal mass that had been evolving over six months. Clinical assessment unveiled the presence of a 2 cm cystic mass located at the anal margin (Fig. 1).

The rectal examination yielded no significant findings.

**Fig. 2:**
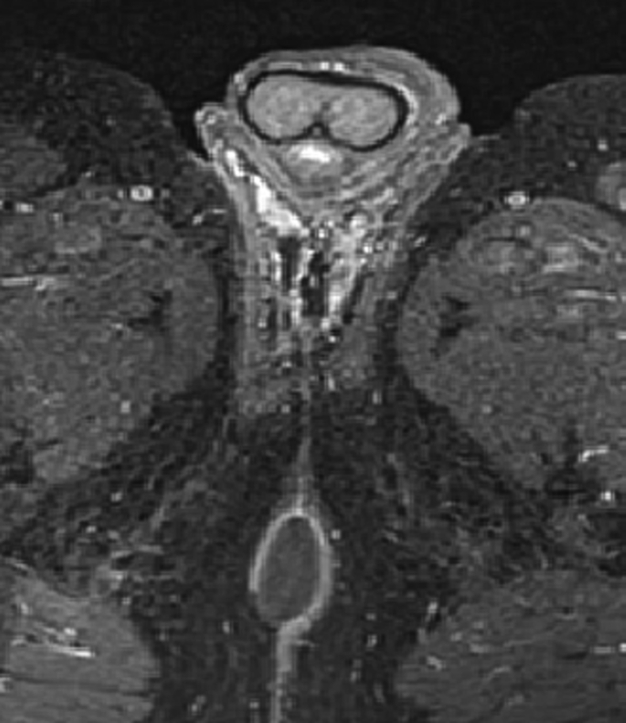
**:** Pelvic MRI images.

Subsequent pelvic Magnetic Resonance Imaging (MRI) was conducted and revealed a well-defined, para-anal cystic mass, measuring 26*15*45 mm in length, exhibiting an oblong configuration, and situated laterally to the right. The mass displayed uniform contours, a pure liquid signal, and a thin wall, enhancing after administration of a contrast agent (Fig. 2).

**Fig. 3:**
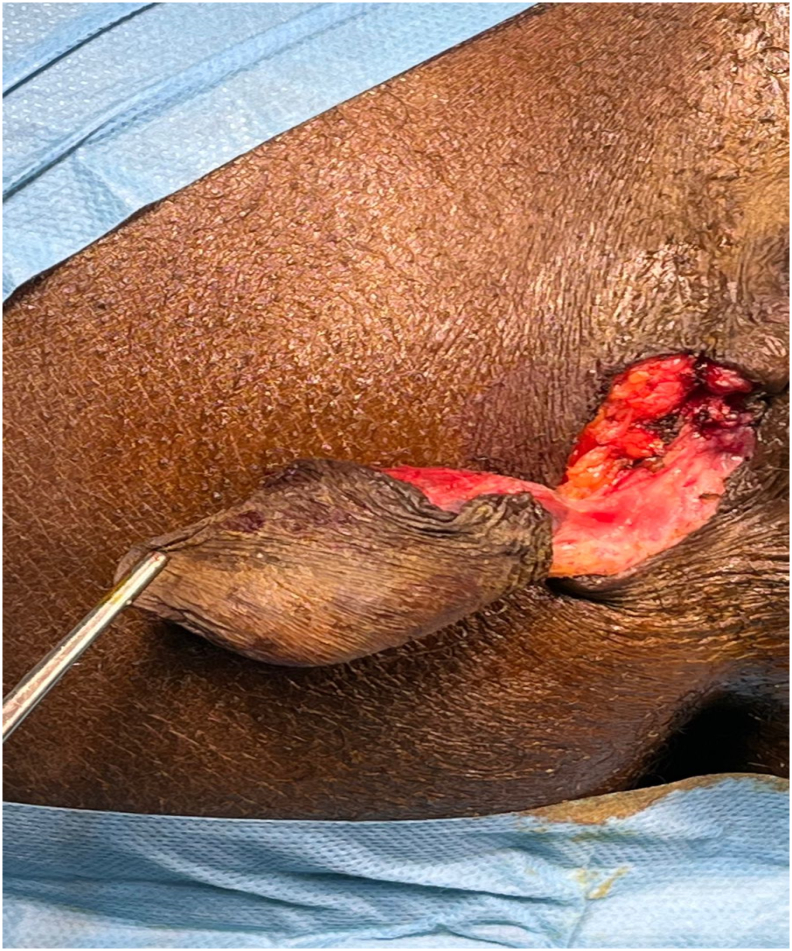
Biopsy Exeresis of the mass.

**Fig. 4:**
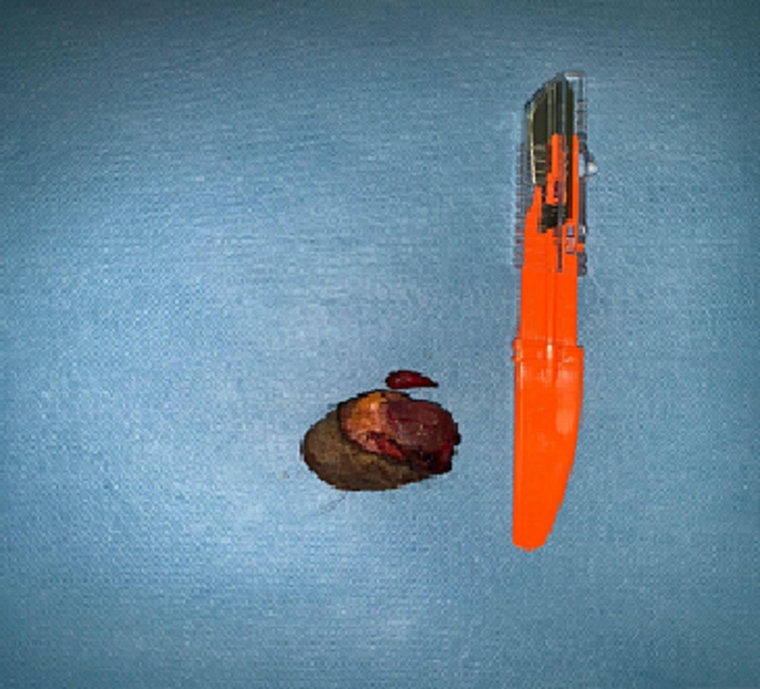
Picture of the specimen A: Histologic examination showing an anal mucosa infiltrated with cystic formation (HE ×10) B: Cystic formation bordered by a double epithelial bed with regular nuclei (HE ×4).

An excisional biopsy procedure was subsequently carried out. (Fig. 3, Fig. 4).

Histological examination revealed a cystic formation bordered by a double epithelial bed with regular nuclei, well-limited by invaginations bearing from the cutaneous surface and extending into the superficial and middle dermis.

These invaginations form multiple branching lumens communicating with the surface.

**Fig. 5:**
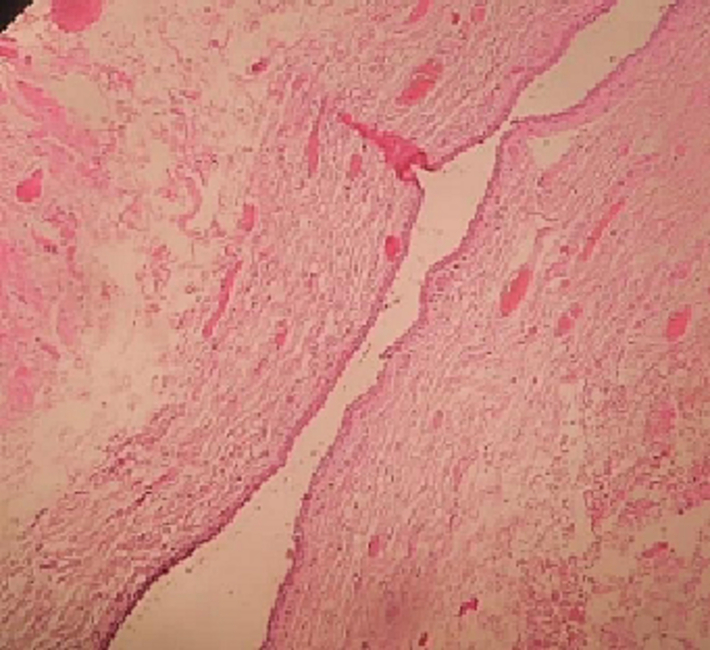
Histopathological picture showing an anal mucosa with a cystic formation delineated by a dual-layered epithelial base characterized by nuclei of regular morphology. This formation was well-contained within invaginations extending from the skin surface into the superficial and middle dermal layers. The invaginations gave rise to multiple branched lumina, which communicated with the skin surface.

They are bordered by a double layer of cells, a liminal layer made up of apocrine-secreting columnar cells and a basal layer made up of cubic cells. The interstitial tissue is made up of a chronic non-specific inflammatory infiltrate, suggesting a syringocystadenoma papiliferum. (Fig. 5).

Postoperative period proceeded smoothly; the patient was discharged the next day.

Currently, 12 months after surgery, the patient is showing no sign of local recurrence.

## 2 Discussion

Syringocystadenoma papiliferum (SCAP) stands as an uncommon adnexal tumor, reported from birth, childhood, puberty to advanced age [5]. It typically presents as a solitary, protruding, nodular mass, often bearing a greyish-brown hue and characterized by either a warty or smooth surface. This distinctive lesion is embedded within an area of skin that exhibits variable prominence, more or less resembling a nipple-like configuration [5,6]. Typically, SCAP lesions range in size between 1 and 3 cm. It is clinically categorized into three distinct types: ‘plate’ (commonly found on the scalp), ‘linear’ (predominantly on the neck or face), and ‘solitary nodular’ (typically encountered on the trunk).

Predominantly, SCAP occurs on the head and neck regions (75 %), followed by the trunk (25 %), and less frequently on the extremities (5 %).

Occasionally, it has been reported in less common sites such as arms, chest, breast, eyelids, armpits, scrotum, inguinal and perineal regions, as well as the external auditory canal, scalp, popliteal fossa [5,[7], [8], [9]].

The clinical presentation of SCAP often lacks specificity, which can lead to diagnostic challenges. Consequently, a definitive diagnosis of syringocystadenoma papiliferum is typically achieved through histopathological examination of the lesion. This tumor is characterized by the proliferation of epithelial structures that manifest both exophytically and endophytically. Its histological features include the presence of tubular and papillary formations, demarcated by a dual-layered arrangement of epithelial cells: an inner layer comprising cylindrical cells and an outer layer characterized by cuboidal cells. The stromal component of the tumor is generally characterized by a preponderance of plasma cells. Additionally, the epidermis may exhibit pseudo-epitheliomatous hyperplasia. It is crucial to differentiate syringocystadenoma papiliferum from papilliferous hidradenoma and adenocarcinoma metastases [10].

Notably, the morphological characteristics of anogenital SCAP appear to closely resemble those observed in other anatomical locations [11]. A cohort study that compared anogenital SCAP with SCAP occurring in other regions confirmed the role of HRAS and BRAF V600 mutations in the pathogenesis of SCAP, irrespective of its location, including the anogenital and gluteal regions [11].

Although the transformation of SCAP into adenocarcinoma or malignant degeneration is exceedingly rare [10,12], the recommended course of action involves complete prophylactic surgical excision, followed by meticulous histological examination. This approach remains the treatment of choice. In instances where surgical excision proves challenging, the CO2 laser represents a viable therapeutic alternative [10].

## 3 Conclusion

Syringocystadenoma papiliferum (SCAP) a rare tumor, and its occurrence in the perineal region is unusual. Histopathological examination of excised tissue samples is essential to establish an accurate diagnosis, to exclude malignant transformation and to differentiate between other possible diagnoses.

## Consent of publication

Written informed consent was obtained from the patient for publication of this case report and accompanying images. A copy of the written consent is available for review by the Editor-in-Chief of this journal on request.

## Ethical approval

Ethical approval is exempt/waived at our institution.

## Funding

N/A.

## Conflict of interest statement

N/A.
